# Event and Comment

**Published:** 1912-10-15

**Authors:** 


### DENTAL REGISTER.

**Vol. LXVI. October 15, 1912. No. 10**

### EVENT AND COMMENT.

FINANCIAL INVESTMENTS. The problem of profitable and safe investment of the savings of the professional man is a difficult one to solve. In these days it would seem that every dentist would have learned that he could not expect either profit or security from the innumerable "get rich" schemes brought to him by mail and solicitous friends and neighbors, we hear almost every day of instances where hard earned savings have been lost through confiding in the fascinating tales told by unscrupulous promoters. We have felt that life insurance, because of its protection and endowment features was one of the most desirable forms of investment for the dentist, and it will probably prove to be in the long run not only the safest but the most profitable. Investments in improved real estate are also to be strongly commended, both of these however require continual and periodic payments or large initial outlays and in some cases are impracticable. The most convenient and safest investments are probably municipal bonds, but these investments are so readily convertible and pay such low rates that when temptations come for "*big payers*" they are as easily exchanged as deposits in the savings bank, and as surely lost as the cash investments. We have recently had our attention directed to the making of investments by dentists in small fruit farms as not only safe but profitable undertakings, not only because they are financially safe but because they offer a means of healthful recreation that is worth more to the dentist than life insurance or money in the bank or bonds in the safety box. This form of recreative investment is the best life insurance, because of its healthful and hygienic opportunities. It is not so convenient perhaps for the city located dentist as for the town or village practitioner; but a half hour train ride will take even the city dentist to the suburbs where a small amount of ground can be bought reasonably, and the plant established with all the necessary health making functions, and it may be with some financial gains also. A small enterprise if carefully planned and religiously developed can not only be made profitable in a few years, but will in time provide a place of refuge for spending the later years of one's life in comfort and easy living. We are aware that failure and disappointment has come from this form of investment in the past, and because of circumstances the best laid farmer's plans may not work out satisfactorily. There is an element of chance in farming as well as in the more speculative investments, but the idea of having something healthful and enticing to set ones mind on and to really live for in the future is calculated to inspire one with an optimistic frame of mind that will react favorably on the drudgery of the routine of office practice. The idea that one can have even a Saturday afternoon or a Sunday at the farm, winters as well as summers is worth a lot to relieve the tension of an exacting practice. Why not get a five or ten acre pleasantly located farm convenient of access and plant it in whatever suits your fancy? A small cottage and barn for a Keeper, who will earn his own pay, and a chance to make a possible home for the future ought to be profitable in many ways. If well managed it should be profitable, and if it becomes tiresome it is always saleable. Lets get back to the farm.

